# Comment on: Over-the-counter sales of high-priority antibiotics are common: results of a global survey

**DOI:** 10.1093/jacamr/dlag247

**Published:** 2026-11-13

**Authors:** Carl Llor, Ana Moragas

**Affiliations:** Institute for Primary Health Care Research Jordi Gol i Gurina (IDIAPJGol), Barcelona, Spain; CIBER de Enfermedades Infecciosas, Instituto de Salud Carlos III, Madrid, Spain; Research Unit for General Practice, Department of Public Health, University of Southern Denmark, Odense, Denmark; Institute for Primary Health Care Research Jordi Gol i Gurina (IDIAPJGol), Barcelona, Spain; CIBER de Enfermedades Infecciosas, Instituto de Salud Carlos III, Madrid, Spain; Jaume I Health Centre, Catalonian Institute of Health, Tarragona, Spain; University Rovira i Virgili, Reus, Spain

To the Editor,

We read with great interest the article describing the global availability of antibiotics without prescription across 37 countries recently published in this journal.^1^ The finding that nearly two-thirds of respondents reported over-the-counter (OTC) access to antibiotics highlights the persistent gap between legislation and real-world practice. This percentage is likely to be even higher, as the information was obtained through a questionnaire rather than more accurate methods, such as the use of mystery shoppers.^2^ Particularly concerning is the reported availability of WHO Watch and Reserve antibiotics, including fluoroquinolones, carbapenems and glycopeptides, without a prescription.^3^ These antibiotics have a high potential to drive antimicrobial resistance and should therefore be preserved through robust antimicrobial stewardship programmes.^4^

The availability of OTC antibiotics is particularly common in low- and middle-income countries, where regulations governing medicine sales are often weak or inadequately enforced, although non-prescription antibiotic dispensing also persists in several high-income countries, including parts of Europe, as mentioned in this manuscript.^5,6^ In many resource-limited settings, community pharmacies represent the first point of contact with the healthcare system because of poor access to primary care, long waiting times and financial or geographical barriers. Consequently, understanding not only the prevalence but also the quality of antibiotic dispensing practices is essential for tackling one of the major drivers of antimicrobial resistance.

While strengthening regulatory enforcement is undoubtedly necessary, focusing exclusively on eliminating all OTC antibiotic sales may neither be feasible nor represent the most effective stewardship strategy.^7,8^ A more pragmatic approach would be to prioritize the prohibition of non-prescription dispensing of Watch and Reserve antibiotics worldwide while allowing carefully regulated access to selected access antibiotics in clearly defined clinical situations. The WHO AWaRe classification was specifically developed to encourage this type of stewardship approach by promoting Access antibiotics as first-line therapy and preserving broader-spectrum agents for situations in which they are genuinely required.^3^

Importantly, not all non-prescription antibiotic supplies should automatically be regarded as inappropriate. In several countries, pharmacists are authorized to prescribe or dispense antibiotics for specific uncomplicated infections within structured clinical pathways. Examples include uncomplicated cystitis, streptococcal pharyngitis confirmed by rapid antigen testing, and chlamydial infection, as well as recurrent uncomplicated urinary tract infections or recurrent genital herpes in patients able to recognize their symptoms. Likewise, point-of-care testing performed in community pharmacies when indicated has been shown to improve antibiotic targeting while reducing unnecessary prescribing. These models differ fundamentally from unregulated OTC sales because they are supported by clinical protocols, pharmacist training and appropriate regulatory oversight.^9^

Community pharmacists are ideally positioned to become key partners in antimicrobial stewardship. Their role should extend beyond dispensing medicines to include patient education, optimization of antibiotic selection and treatment duration, use of point-of-care diagnostics where appropriate and referral of patients with complicated infections. Achieving this expanded role requires enhanced antimicrobial stewardship training, continuing professional development, supportive legislation and closer integration of pharmacists within primary healthcare teams. Regulatory policies should also promote rational antibiotic use by encouraging exact quantity dispensing, smaller pack sizes, shorter evidence-based treatment courses and restricting non-prescription access to broad-spectrum antibiotics while favouring narrow-spectrum Access agents for appropriate indications.^9,10^

Ultimately, the success of regulatory interventions should be assessed not only by reductions in OTC antibiotic sales but also by improvements in antibiotic use and antimicrobial resistance. Future surveillance should therefore collect information on the specific antibiotics dispensed, their clinical indications, pharmacists’ decision-making and resistance trends to better inform effective antimicrobial stewardship policies.

## References

[1] M’ikanatha NM, Yin X, Iskander K et al Over-the-counter of high-priority antibiotics are common: results of a global survey. JAC Antimicrob Resist 2026; 8: dlag083. 10.1093/jacamr/dlag083 PMC13197841 42179901

[2] Björnsdottir I, Granas AG, Bradley A et al A systematic review of the use of simulated patient methodology in pharmacy practice research from 2006 to 2016. Int J Pharm Pract 2020; 28: 13–25. 10.1111/ijpp.12570 31397533

[3] World Health Organization . AWaRe classification of antibiotics for evaluation and monitoring of use. 2023. https://www.who.int/publications/i/item/WHO-MHP-HPS-EML-2023.04

[4] Dyar OJ, Huttner B, Schouten J et al What is antimicrobial stewardship? Clin Microbiol Infect 2017; 23: 793–8. doi: 10.1016/j.cmi.2017.08.026 28882725

[5] Auta A, Hadi MA, Oga E et al Global access to antibiotics without prescription in community pharmacies: a systematic review and meta-analysis. J Infect 2019; 78: 8–18. 10.1016/j.jinf.2018.07.001 29981773

[6] Guinovart MC, Figueras A, Llop JC et al Obtaining antibiotics without prescription in Spain in 2014: even easier now than 6years ago. J Antimicrob Chemother 2015; 70: 1270–1. 10.1093/jac/dku526 25558070

[7] Llor C, Benkő R, Bjerrum L. Global restriction of the over-the-counter sale of antimicrobials: does it make sense? Front Public Health 2024; 12: 1412644. 10.3389/fpubh.2024.1412644 PMC11251895 39022420

[8] Guinovart MC, Figueras A, Llor C. Selling antimicrobials without prescription—far beyond an administrative problem. Enferm Infecc Microbiol Clin 2018; 36: 290–2. 10.1016/j.eimc.2016.10.006 27866752

[9] Lambert M, van Dijk L, Benko R et al The role of the community pharmacist in antibiotic use—a commentary on current status and future perspectives. Explor Res Clin Soc Pharm 2025; 20: 100652. 10.1016/j.rcsop.2025.100652 PMC12446664 40980189

[10] Saha SK, Barton C, Promite S et al Knowledge, perceptions and practices of community pharmacists towards antimicrobial stewardship: a systematic scoping review. Antibiotics (Basel) 2019; 8: 263. 10.3390/antibiotics8040263 PMC6963969 31842511

